# Microglial ApoD‐induced NLRC4 inflammasome activation promotes Alzheimer's disease progression

**DOI:** 10.1002/ame2.12361

**Published:** 2024-03-23

**Authors:** Yaliang Yu, Jianzhou Lv, Dan Ma, Ya Han, Yaheng Zhang, Shanlong Wang, Zhitao Wang

**Affiliations:** ^1^ Department of Neurology The Second Affiliated Hospital of Henan University of Science and Technology Luoyang P. R. China; ^2^ Clinical Lab The Second Affiliated Hospital of Henan University of Science and Technology Luoyang P. R. China

**Keywords:** Alzheimer's disease, ApoD, microglia, NLRC4 inflammasome

## Abstract

**Background:**

Alzheimer's disease (AD) is a progressive neurodegenerative disease with no effective therapies. It is well known that chronic neuroinflammation plays a critical role in the onset and progression of AD. Well‐balanced neuronal‐microglial interactions are essential for brain functions. However, determining the role of microglia—the primary immune cells in the brain—in neuroinflammation in AD and the associated molecular basis has been challenging.

**Methods:**

Inflammatory factors in the sera of AD patients were detected and their association with microglia activation was analyzed. The mechanism for microglial inflammation was investigated. IL6 and TNF‐α were found to be significantly increased in the AD stage.

**Results:**

Our analysis revealed that microglia were extensively activated in AD cerebra, releasing sufficient amounts of cytokines to impair the neural stem cells (NSCs) function. Moreover, the ApoD‐induced NLRC4 inflammasome was activated in microglia, which gave rise to the proinflammatory phenotype. Targeting the microglial ApoD promoted NSC self‐renewal and inhibited neuron apoptosis. These findings demonstrate the critical role of ApoD in microglial inflammasome activation, and for the first time reveal that microglia‐induced inflammation suppresses neuronal proliferation.

**Conclusion:**

Our studies establish the cellular basis for microglia activation in AD progression and shed light on cellular interactions important for AD treatment.

## Graphical abstract

We found that NLRC4 inflammasome was activated in microglia releasing IL6 and TNF‐α to impair the hippocampal neural stem cells (NSCs) self‐proliferation. ApoD contributed to activating NLRC4 inflammasome in microglia. Targeting the ApoD provides beneficial effects on NSCs self‐proliferation and mature neurons survival.

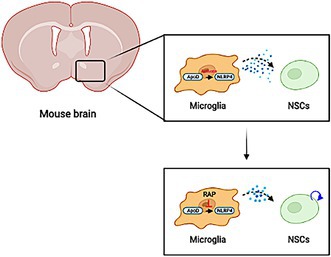

## 1 INTRODUCTION

Alzheimer's disease (AD) is a progressive disorder, causing neurons to degenerate, which is the most common cause of dementia.^1^ AD patients present an age‐related continuous decline in memory and cognition that disrupts their ability to function independently. As disease progresses, AD patients develop severe memory impairment and lose the ability to carry out daily activities.^2^ In the advanced stages of AD, complications such as dehydration, malnutrition or infection result in death. Current medications for AD may temporarily improve symptoms or ease the rate of decline, but there is no effective treatment for AD that protects neurons from constant injury.^3^ Therefore, a deeper understanding of the biological behaviors and molecular basis of AD is required for generating the therapeutic strategies.

At its core, AD profoundly affects the lives of those diagnosed with it. Initially, individuals may experience mild memory lapses and confusion, but as the disease progresses, it exacts an emotional and physical toll that is difficult to overstate.^4^ Patients often lose their ability to recognize loved ones, remember significant life events, or even engage in coherent conversation.^5^ This cognitive decline robs them of their independence, self‐identity, and dignity, making Alzheimer's one of the most feared diagnoses in the world.

AD manifests histologically as the parenchymal deposition of amyloid‐beta (Aβ) plaques and neuroinflammation.^6^, ^7^ Recently, many studies have reported immunity derangement contributing to AD onset and disease progression.^8^ Under such conditions, brain‐resident microglia acquire proinflammatory activity, which has been shown to correlate with disease escalation.^9^ Microglia, the resident myeloid cells in central nervous system (CNS) and a major component of the brain immune system, play an essential role in neuronal homeostasis and regulate multiple pathogeneses of disorders such as neurodegenerative diseases and tumors.^10^ Recently, numerous studies have reported that brain‐resident microglia can be distinguished from bone marrow‐derived macrophages.^11^, ^12^ Under homeostatic conditions, microglia originate from hematopoietic stem cells in the yolk sac but not from bone marrow.^13^ In the pathogenesis of AD, microglia are concentrated around amyloid plaques and affect the function of neurons via releasing a wide range of cytokines.^14^, ^15^ Therefore, targeting the microglia presents a novel therapeutic approach to this difficult‐to‐treat disorder. This includes developing drugs that can shift microglia towards a more neuroprotective and anti‐inflammatory state, as well as enhancing their ability to clear abnormal protein aggregates.^16^ Several experimental therapies that target microglial dysfunction are being investigated as a potential strategy for slowing or halting the progression of AD.

Recently, inflammasomes have been recognized for their roles in neurodegenerative disorders, neuroinflammation, tumorigenesis and host defense against pathogen invasion.^17^, ^18^, ^19^ Inflammasomes assemble in response to pathogenesis or tissue damage by the Nod‐like receptor protein (NLR) and are absent in melanoma 2 (AIM2)‐like receptors (ALR).^20^ Lots of proinflammatory cytokines such as IL1β, IL18, IL33, and pyroptosis are induced by inflammasome activation.^21^ They regulate innate immunity particularly by acting as the platforms for activation of Caspase‐1, Caspase‐8, Caspase‐11 and IL‐1R‐associated kinases (IRAK). A role of the innate immune system in driving neuroinflammation has been identified in the etiology of AD. Previously, the best‐studied inflammasome, the NLRP3 inflammasome, was found to be activated in neurons of AD brain, producing a set of cytokines to promote inflammatory reactions.^22^ However, whether microglia are involved in inflammasome activation associated with AD progression is poorly understood. Recent studies indicate that lipid metabolism is linked with inflammasome functions. Krasemann et al. identified the TREM2‐ApoE pathway as a major regulator of the microglia phenotype in neurodegenerative diseases and suggested that targeting this pathway could restore homeostatic microglia.^23^ Our study reveals that, in addition to ApoE, ApoD‐induced NLRC4 inflammasome is extensively activated in microglia of AD patient brains, along with the release of significant amounts of IL6 and TNF‐α, a decline in the proliferation of neural stem cells (NSCs) and promotion of apoptosis of neurons. Inhibition of ApoD‐induced inflammasomes proves beneficial, protecting NSC self‐renewal and thereby opening a new avenue for therapeutic intervention.

## 2 METHODS

### 2.1 Patients and samples

Serum specimens were collected from 62 AD patients (81.2 years, age range 57–93 years) and 6 healthy control subjects at the Department of Neurology in the Second Affiliated Hospital of Henan University of Science and Technology between October 2013 and September 2019. The diagnosis of AD was based on the NINCDS–ADRDA research criteria.^24^ None of patients had received AD associated treatment before blood collection. Patient information including the general characteristics, stages and prognosis of the disease was obtained from the medical records or outpatient follow‐up records and is listed in Table 1. All brain tissues of AD patients or healthy controls were harvested from six donors. Subsequently the samples were immediately frozen with liquid nitrogen and stored until further study. The aforementioned procedures were approved by the Research Ethics Committee of The Second Affiliated Hospital of Henan University of Science and Technology. All the methods were carried out in accordance with relevant guidelines and regulations. The study is reported in accordance with ARRIVE guidelines. All patients provided written informed consent according to the *Declaration of Helsinki*.

**TABLE 1:** Characteristics of patients with AD (62 cases).

Items	No. (%)	OR
Gender		0.66
Male	24 (38.71)	
Female	38 (61.29)	
Age (y)		1.39
<60	2 (3.23)	
60–80	23 (37.10)	
>80	37 (59.68)	
Prognosis		0.88
Alive	49 (79.03)	
Dead	13 (20.97)	
Stage		1.52
MCI	28 (45.16)	
AD	34 (54.84)	
IL1β		0.87
<2500	30 (48.39)	
>2500	32 (51.61)	
IL18		0.71
<3000	28 (45.16)	
>4000	34 (54.84)	
IL6		2.09
<4000	25 (40.32)	
>4000	37 (59.68)	
TNF‐α		1.68
<3000	23 (37.10)	
>3000	39 (62.90)	

Abbreviations: AD, Alzheimer's disease; MCI, mild cognitive impairment.

### 2.2 Mice

C57/BL6 wild‐type (WT) mice were acquired from Jackson Laboratory. The 3 × Tg AD mouse models were purchased from Beijing Vitalriver Co., Ltd (Beijing, China). All animals were maintained in the Laboratory Animal Facility at The Second Affiliated Hospital of Henan University of Science and Technology.

Experiments were performed in accordance with procedures approved by the Animal Care Committee (2023‐10‐19‐035).

### 2.3 Cells

The microglia cell line BV2 was purchased from the Institute of Basic Medical Sciences Peking Union Medical College. The BV2 cells were authenticated using cellular morphology and cultured in DMEM (Gibco) supplemented with 10% FBS (Gibco) and penicillin/streptomycin (1%) at 37°C in a humidified incubator with 5% CO_2_. To obtain the NSCs, murine hippocampal tissues were dissociated into a single cell suspension for further FACS sorting using the CD133 surface marker. The harvested cells were cultured in Neurobasal medium (Gibco) containing B27 (2%), EGF (50 ng/mL), bFGF (20 ng/mL), L‐glutamine (1%) and penicillin/streptomycin (1%).^25^ To collect the neurons, the cortexes of fetal or adult mice were digested with 0.25% trypsin and the dissociated cells were suspended at 1 × 10^7^/mL in Neurobasal medium supplemented with B27 (2%), L‐glutamine (1%) and penicillin/streptomycin (1%) to isolate the neurons (bound part) and glia (suspended part).

### 2.4 Immunohistochemistry

Tissues were dehydrated utilizing an ethanol alcohol gradient followed by incubation with 100% alcohol three times for 5 min. The paraffin‐embedded block was sliced into 5 μm sections and washed in a 40°C water bath for 15 min. Sections were fixed in 4% PFA (Invitrogen) at 4°C overnight. The sections were heated in 5% FBS (Gibco) at 75°C for 30 min to reduce non‐specific background contamination. Briefly, to investigate the expression of Aβ, IBA1 and NLRC4 in brain tissues, the prepared sections were stained with antibodies against Aβ (Ab120851, Abcam, 1:100), IBA1 (Ab5076, Abcam, 1:100), NLRC4 (CST12421, Cell Signaling Tech, 1:100), Caspase‐1 (CST24232, Cell Signaling Tech, 1:100) and ApoD (Ab191275, Abcam, 1:200) at 4°C overnight. Then sections were washed with PBS and incubated with the secondary antibodies for 1 h at room temperature. The scores for stained cells were calculated by counting the average number of positive cells in 500 nuclei in four random light high‐power magnifications (400 X). The average IHC scores for Aβ, IBA1 and NLRC4 were 3, 4 and 4, respectively. Histopathological diagnoses were independently confirmed by two pathologists based on the WHO criteria.^26^

### 2.5 In situ hybridization

DIG‐UTP‐labeled probes for *APOD* were added to this mixture on frozen tissue sections, and then hybridization was performed at 60°C for 48 h. After hybridization, sections were incubated overnight at 4°C with the alkaline phosphatase‐conjugated antibody against DIG. Bound probe was visualized by incubating sections in NBT/BCIP at 4°C overnight in the dark.

### 2.6 ELISA

The concentration of IL1β, IL6, IL18 and TNF‐α was examined using the ELISA Kit (Sigma). Duplicate 100 μL aliquots of cell culture supernatant or sera were added and incubated at 37°C for 90 min. After washing 3 times, the indicated antibodies were added and incubated at 37°C for 60 min. After washing three times, 100 μL of the indicating antibodies from the ELISA kits were added and incubated at 37°C for 60 min. After the color reaction with the TMB substrate for 15 min, absorbency was detected at 450 nm in a microplate reader.

### 2.7 Western blot analysis

Tissues and cells were lysed in splitting buffer (pH = 7.4) containing protease‐inhibitor cocktail. Whole proteins were separated in 10% SDS‐PAGE and transferred to a PVDF membrane. Membranes were subsequently incubated with antibodies against IL1β, IL6, IL18, TNF‐α, ApoD, Caspase‐1, NLRC4 and β‐actin overnight at 4°C, and then probed with secondary antibodies for 1 h at room temperature. All the antibodies were diluted at 1:500 except for β‐actin, which was diluted at 1:3000.

### 2.8 RT‐PCR

RNA was isolated using Trizol reagent according to the manufacture's procedures (Invitrogen). cDNA was synthesized using the iScript reverse transcription kit. Quantitative levels of mRNA were determined using SYBR‐green on the Mx3000P QPCR system and normalized for expression of GAPDH mRNA.

### 2.9 Flow cytometry analysis

Flow cytometry (FCM) analysis was performed based on the manufacture instructions. Briefly, cells were diluted to 1 × 10^5^/mL, dissociated in 1 X binding buffer, then stained with Annexin V‐FITC (Biolegend) and propidium iodide (PI) in the dark at room temperature for 20 min and finally analyzed using FCM. The apoptosis rate was calculated as the percentage of double Annexin V‐FITC^+^/PI^+^ cells in the total cell sample.

### 2.10 Lentivirus production

Lentivirus was prepared using shRNA of NLRC4, shRNA of ApoD and scrambled shRNA (Sigma), and pMD2.G and psPAX2 (Addgene) plasmids according to the standard protocol. Briefly, 293T packaging cells were transfected with 2 M CaCl_2_ and incubated for 8 h before replacing the culture media with 10 mL of DMEM complete medium with 2–5 mM sodium butyrate. The virus was collected at 48 and 72 h following the transfection.

### 2.11 MTT assay

NSCs (1000 cells/well) were seeded into 96‐well plates for 24 h (37°C, 5% CO_2_). Cell viability was evaluated by performing the MTT assay. Briefly, 0.5 mg MTT reagent was added to each well for 4 h of incubation. After removing the supernatant, 100 μL DMSO (J&K) was added to each well and incubated for 10 min. The purple mixture in each well was then measured at 490 nm using the Polarstar Optima microplate reader. The absorbance was tested at 660 nm to measure background signals due to cell debris and excess coupling reagent.

### 2.12 Statistical analysis

Data were presented as the means ± standard deviation. Significant differences were determined with Student's *t* test and the Mann–Whitney test using SPSS19.0 and GraphPad Prism8.0. The unpaired *t* test was used for comparison between two groups, and comparison of mean values between multiple groups was evaluated by one‐way ANOVA followed by Student–Newman–Keuls post hoc test. The functional experiments were performed at least three times.^27^ The correlation between *NLRC4* mRNA and *IL6* or *TNFA* mRNA expression was analyzed using Pearson correlation. Logistic regression was performed to analyze the clinical risk factors. *p* < 0.05 was considered to indicate a significant difference.

## 3 RESULTS

### 3.1 Microglia are activated in AD brains, resulting in neuroinflammation

**FIGURE 1:**
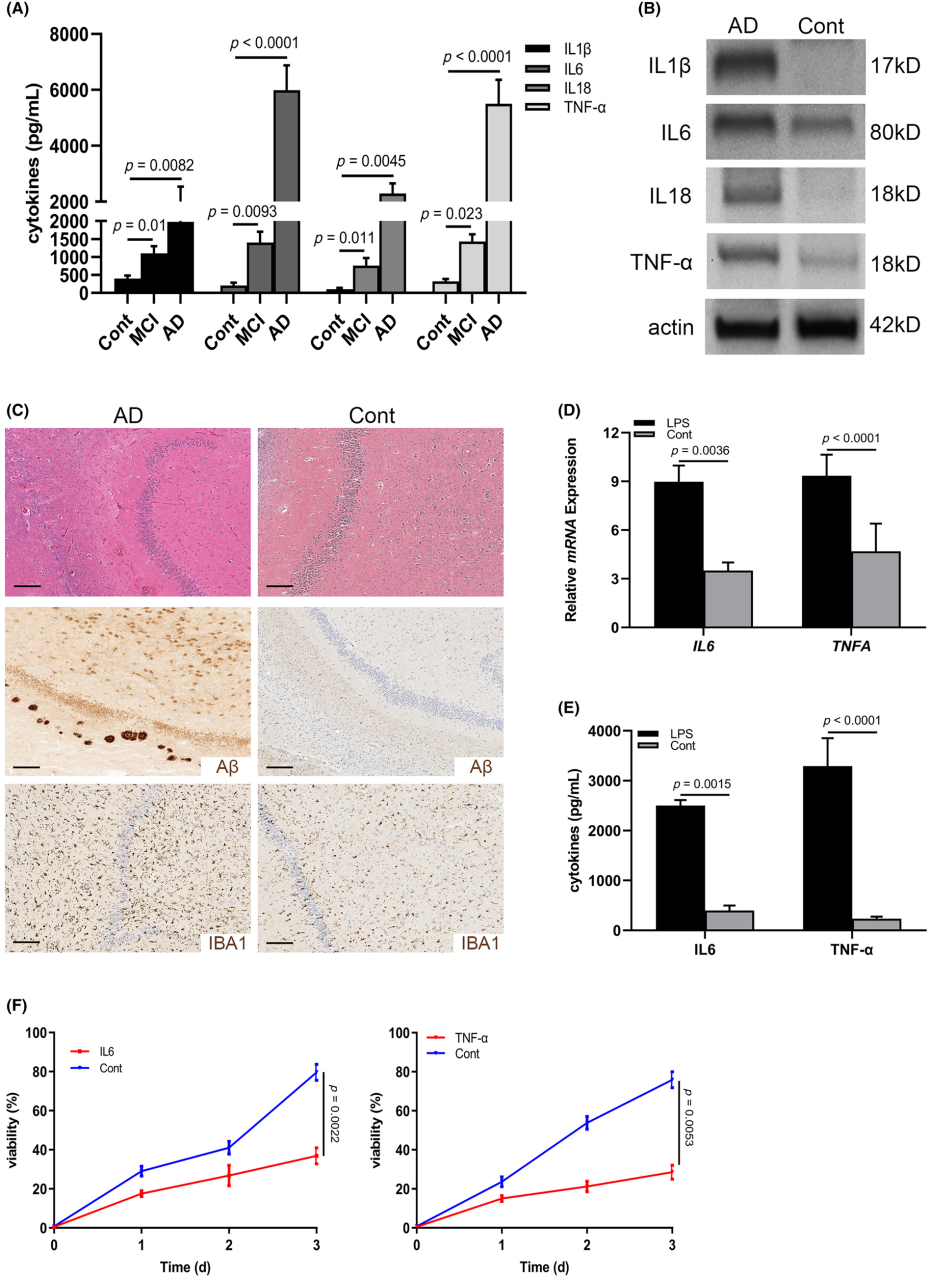
Microglia are activated in AD brains. (A) The expression of IL1β, IL6, IL18 and TNF‐α in the sera from healthy controls (*n* = 6), MCI (*n* = 28) and AD (*n* = 34) patients examined by ELISA. (B) The expression of IL1β, IL6, IL18 and TNF‐α in human healthy brains and AD brains tested by western blot. β‐Actin protein was chosen as the internal control. (C) Representative images of IHC staining of Aβ and IBA1 in hippocampus of AD patients (*n* = 3) and healthy controls (*n* = 3; bar, 10 μm). (D) mRNA expression of *IL6* and *TNFA* in cerebra tissues from LPS‐treated mice (*n* = 6) and controls. (E) The expression of IL6 and TNF‐α in supernatant of BV2 cells treated with LPS (*n* = 3). (F) MTT assay showing the proliferation of NSCs treated with IL6 and TNF‐α (*n* = 6). All the experiments were performed at least three times.

Neuroinflammation has been identified as one important characteristic of AD.^28^ To observe the inflammation in patients with AD, a total of 6 healthy controls and 62 AD cases were included in the current cohort: 28 (45.16%) patients suffered from mild cognitive impairment (MCI) and 34 (54.84%) were in AD stage (Table 1). Sera were collected from these patients to examine the levels of well‐established inflammatory factors including IL1β, IL6, IL18 and TNF‐α. As shown in Figure 1A, all the cytokines were elevated in both MCI and AD patients, and were progressively increased in the AD stage patients. Furthermore, IL6 and TNF‐α were most significantly increased in the AD stage patients (Figure 1A and Table 1). As evidenced by Logistic regression analysis, the expression of IL6 and TNF‐α, as well as the parameters of age and AD stage, affected the prognosis, suggesting that they could be the valuable prognostic indicators for patients with AD (Table 1). Similarly, the immunoblotting assay indicated higher levels of IL1β, IL6, IL18 and TNF‐α in AD brain tissues (Figure 1B). Previously, activated microglia have been reported to be a common pathological characteristic of neuroinflammation.^29^ Hippocampus is a critical region for memory and is affected by cognitive decline in elderly people as well as AD patients.^30^ Accordingly, to detect the activation of microglia in the hippocampus of AD brains, we examined the expression of IBA1 and Aβ by IHC in human donor samples. A large number of microglia (IBA1^+^) were present across the Alzheimer's hippocampus compared with the normal control, which correlated positively with the expression of Aβ (Figure 1C). To confirm inflammation in brain tissues after stimulation, we used the well‐verified lipopolysaccharide (LPS) method to treat the mice for 3 days and harvested the cerebra to examine the mRNA levels of *IL6* and *TNFA*. As displayed in Figure 1D, *IL6* and *TNFA* mRNA expression were observed to be upregulated in the LPS‐stimulated mice, demonstrating that LPS could induce neuroinflammation. To further identify the role of microglia in production of IL6 and TNF‐α, BV2 cells were cultured with LPS stimulation for 48 h and the supernatant was collected to examine the concentration of IL6 and TNF‐α. As expected, they were significantly increased in the LPS treatment group (Figure 1E), which suggested that activated microglia could provide these specific inflammatory factors. With the goal of identifying the effects of IL6 and TNF‐α on the proliferation of NSCs, NSCs purified from hippocampus were cultured with IL6 or TNF‐α for 48 h. Subsequent MTT assays showed that self‐proliferation was dramatically impaired by the two cytokines (Figure 1F). Taken together, these data suggest that activated microglia in AD hippocampus attenuate the self‐renewal of NSCs via IL6 and TNF‐α.

### 3.2 NLRC4 inflammasome is activated in microglia of AD brains

**FIGURE 2:**
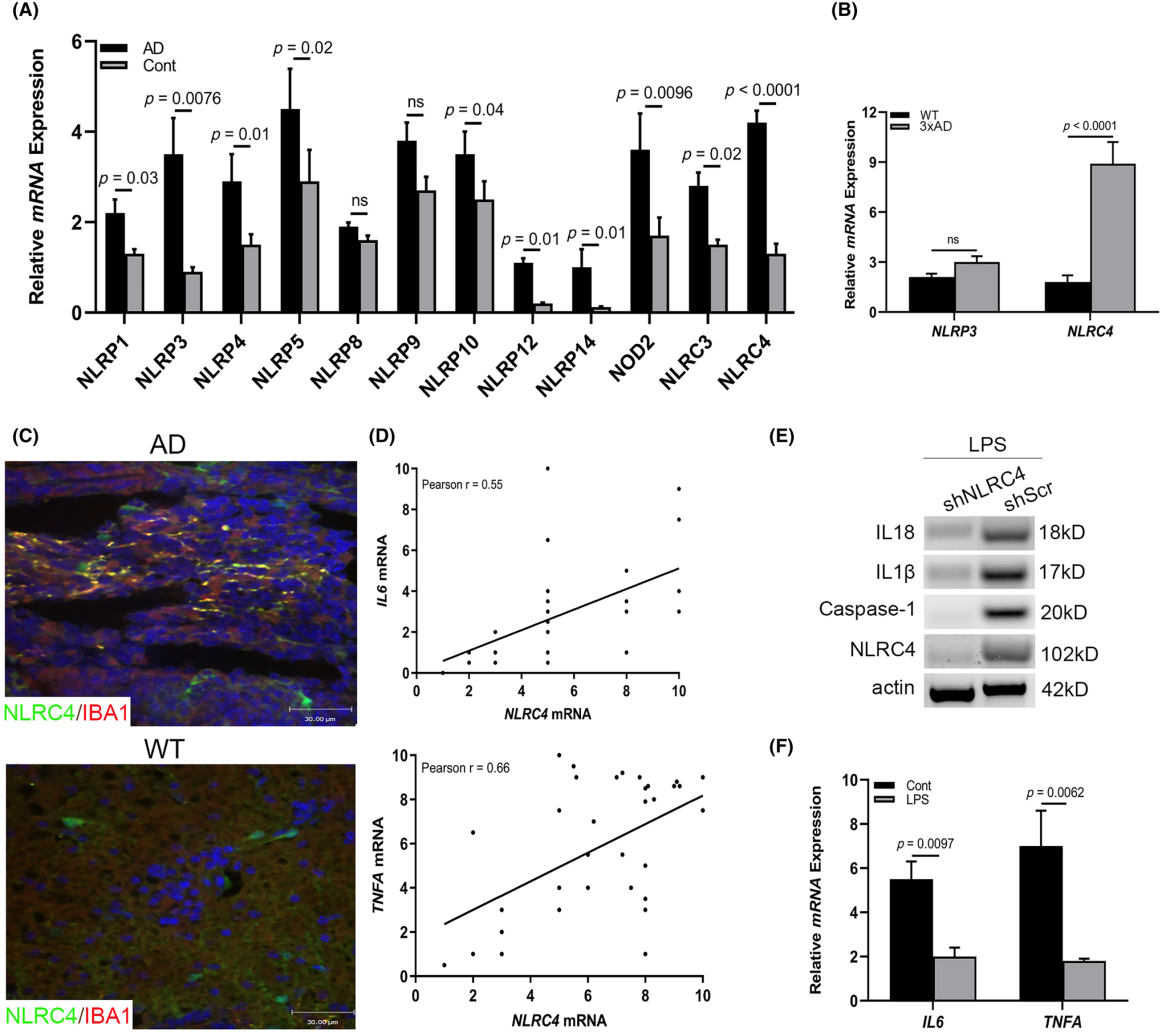
NLRC4 inflammasome is activated in patients with AD. (A) RT‐PCR analysis showing the inflammasome‐associated gene expression (*NLRPs* and *NLRCs)* in human healthy cerebra (*n* = 3) and AD cerebra (*n* = 3) tissues. (B) RT‐PCR analysis showing *NLRP3* and *NLRC4* mRNA expression in microglia of AD and WT mice (*n* = 6). (C) IHC staining of NLRC4 in healthy control cerebra (*n* = 3) and AD cerebra (*n* = 3; bar, 10 μm). (D) Pearson analysis showing the correlation between *NLPC4* mRNA and *IL6* or *TNFA* mRNA. (E) Western blot analysis showing the expression of NLRC4, Caspase‐1, IL1β and IL18 in BV2 cells. (F) RT‐PCR analysis showing *IL6* and *TNFA* mRNA expression in BV2 cells infected with shNLRC4 after LPS stimulation (*n* = 6). All the experiments were performed at least three times.

Having identified microglia activation in AD brains, we next sought to detect the molecular mechanism of the microglial proinflammatory phenotype. Accumulating previous studies have reported that inflammasome activation contributes to releasing a wide variety of cytokines such as IL1β, IL18 and TNF‐α in the absence of cell death.^20^, ^31^ To detect the specific activation of inflammasomes during progression of AD, RT‐PCR analysis was performed in the cerebra tissues of AD patients and healthy control donors, which indicated that *NLRP3* and *NLRC4* mRNA levels were significantly increased in AD patients in contrast to the control group (Figure 2A). The best‐studied inflammasome, the NLRP3 inflammasome, has been reported to be activated in neurons with α‐synuclein pathology and dopaminergic neurodegeneration in mice.^22^ However, whether microglia undergo NLRC4 inflammasome activation to promote neurodegeneration is poorly understood. To address this, microglia were collected from 3 × Tg AD mice cerebra tissues and the mRNA levels of *NLRP3* and *NLRC4* were examined by RT‐PCR test. As shown in Figure 2B, there were significant differences in *NLRC4* mRNA expression. Similarly, a significant population (31.2%) of microglia expressed NLRC4 in the hippocampus of AD mice (Figure 2C). As shown in Figure 1B, the expression of IL1β and IL18, markers of inflammasome activation, were also significantly increased in AD brain tissues. In order to link NLRC4 inflammasome activation with the proinflammatory phenotype, we further analyzed the RT‐PCR data and found a positive correlation between *NLRC4* and *IL6/TNFA* mRNA expression (Figure 2D). As shown in Figure 1E, microglia produced increased amounts of IL6 and TNF‐α after LPS stimulation. To explore the role of NLRC4 in interleukin production, we generated BV2 cells using a lentiviral approach (shRNA) to reduce the expression of NLRC4. After NLRC4‐deficient cells were treated with LPS for 72 h, an immunoblotting assay was performed, which showed decreased levels of NLRC4, Caspase‐1, IL1β and IL18, thus indicating the inactivation of microglial NLRC4 inflammasomes (Figure 2E). Furthermore, the *IL6* and *TNFA* mRNA expression were observed to be downregulated demonstrating inflammation phenotype was attributed to NLRC4 inflammasome activation (Figure 2F). Collectively, these data strongly suggest that microglial NLRC4 inflammasome activation contributes to the proinflammatory features.

### 3.3 ApoD is required for microglial NLRC4 inflammasome activation

**FIGURE 3:**
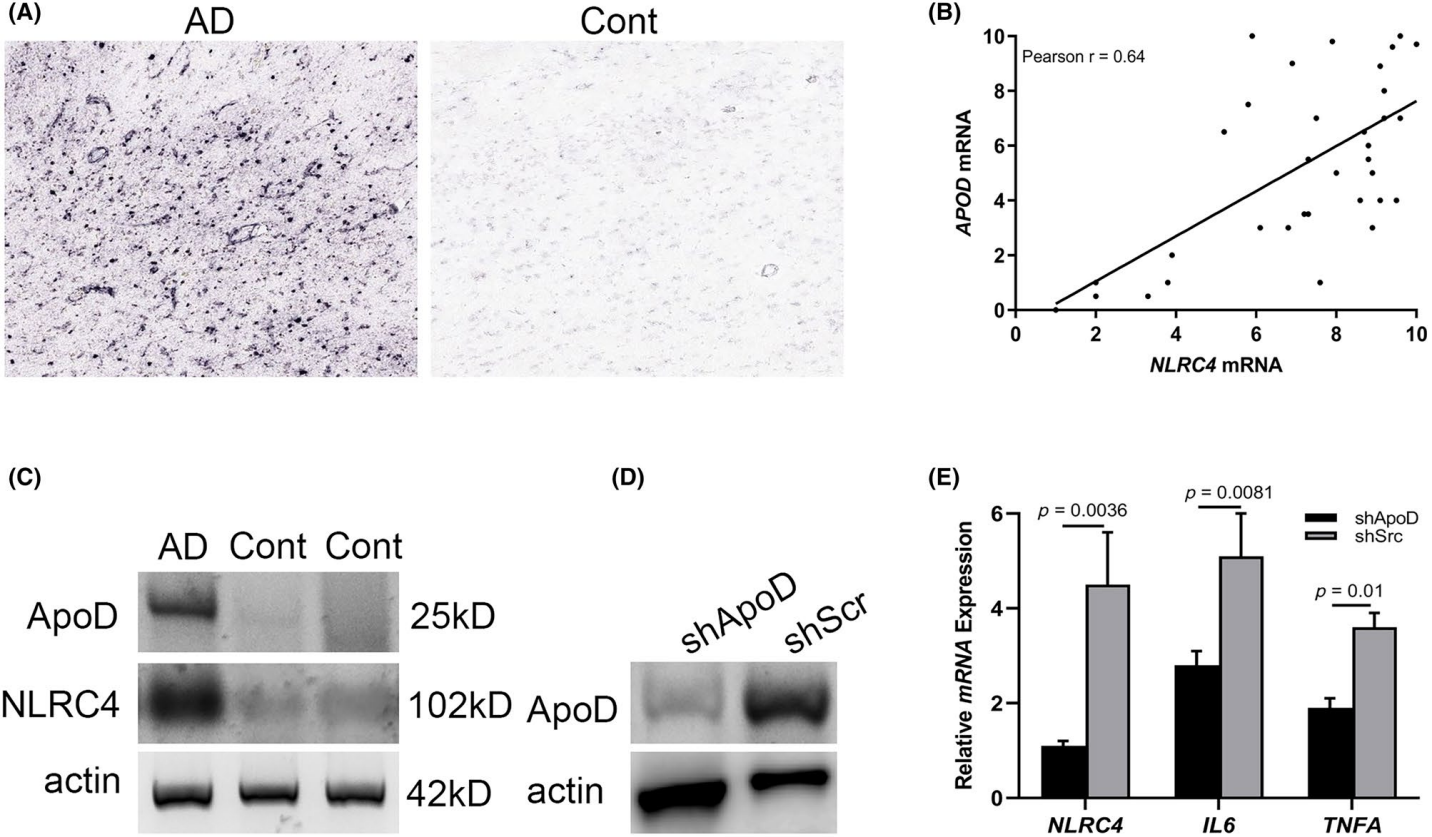
ApoD is require for microglial NLRC4 inflammasome activation. (A) ISH staining of ApoD in healthy control cerebra (*n* = 3) and AD cerebra (*n* = 3; bar, 10 μm). (B) Pearson analysis showing the correlation between *APOD* and *NLRC4 mRNA*. (C) Immunoblotting assay showing the expression of ApoD and NLRC4 expression in brain tissues of AD patients and controls. β‐Actin protein was chosen as the internal control. (D) Immunoblotting assay showing the expression of ApoD in BV2 cells infected with shApoD and shSrc. (E) RT‐PCR analysis showing *NLPC4, IL6* and *TNF1* mRNA expression in BV2 cells infected with shApoD after LPS stimulation (*n* = 6). All the experiments were performed at least three times.

Previously, ApoD has been identified as being downregulated in microglia during development. Upon neurodegeneration or neuroinflammation, ApoD as a key molecule, broadly activating diverse pathways.^32^, ^33^ The expression of microglial ApoD prompted us to ask whether it could induce NLRC4 activation. ISH staining of *APOD* was diffusedly observed in cerebra of AD patients, supporting the evidence linking ApoD with Alzheimer's pathogenesis (Figure 3A). Moreover, a strong correlation between *APOD* and *NLRC4* expression was found based on RT‐PCR and immunoblotting analysis of human AD brain tissues (Figure 3B,C). To further establish the role of ApoD in NLRC4 inflammasome activation, BV2 cells were infected with shApoD or scrambled shRNA to delete ApoD expression (Figure 3D). After treating the ApoD‐deficient BV2 cells with LPS, the RT‐PCR assay showed the suppression of *NLRC4, IL6* and *TNFA* in AD tissue compared with the controls (Figure 3E). Thus, the above data indicate that ApoD confers proinflammatory properties on microglia through NLRC4 inflammasomes.

### 3.4 Targeting the ApoD improves the neuronal function

**FIGURE 4:**
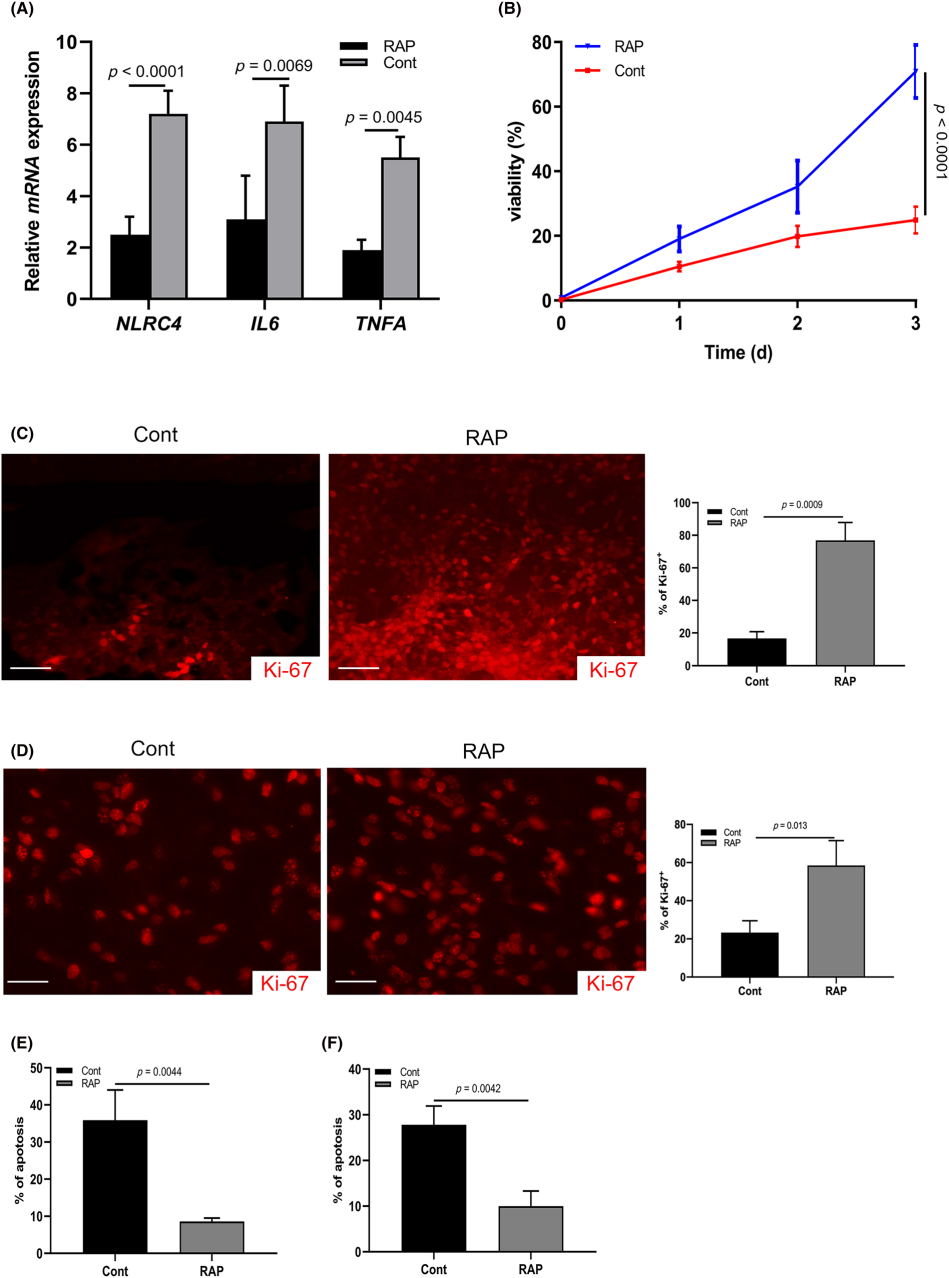
Blockage of ApoD promotes the NSCs self‐proliferation. (A) RT‐PCR analysis showing *NLPC4, IL6* and *TNFA* mRNA expression in BV2 cells treated with RAP or PBS after LPS stimulation (*n* = 3). (B) MTT assay showing the viability of NSCs cultured in the supernatant of LPS‐stimulated BV2 cells with or without RAP treatment (*n* = 3). (C) Immunostaining for Ki‐67 in NSCs co‐cultured with activated BV2 cells with RAP or PBS treatment (*n* = 3). (D) Immunostaining for Ki‐67 in NSCs co‐cultured with activated primary microglia with RAP or PBS treatment (*n* = 3). (E) FCM analysis showing the apoptosis of neurons cultured in the supernatant of LPS‐stimulated BV2 cells with or without RAP treatment (*n* = 3). (F) FCM analysis showing the apoptosis of neurons cultured in the supernatant of microglia from a murine AD model with or without RAP treatment (*n* = 3). All the experiments were performed at least three times.

The above data established that activated microglia contributed to AD progression. Finally, we investigated whether neuronal injury could be inhibited by targeting ApoD‐induced NLRC4 inflammasomes. Receptor‐associated protein (RAP) treatment (20 μg/μL) for 48 h was utilized to block the ApoD in BV2 cell cultures. Following LPS (1 μg/mL) stimulation, *NLRC4, IL6* and *TNFA* mRNA expression was dramatically suppressed by blockage of ApoD (Figure 4A). Subsequently, the supernatant of LPS‐stimulated BV2 cell cultures was harvested and mixed 1:1 with NSCs cultures. MTT assay of the mixed culture showed that NSCs were powerfully suppressed; proliferation was inhibited by 70% in the untreated group compared with the RAP treatment group (Figure 4B). Furthermore, we co‐cultured the NSCs with LPS‐stimulated BV2 cells or primary microglia collected from P4 mice with or without RAP treatment (20 μg/μL) for 48 h. The Ki‐67 index was examined by immunostaining assay and showed a decreased level in the control group (Figure 4C,D). Most NSCs were actively proliferating (Ki‐67^+^) after RAP treatment (Figure 4C,D). These data suggest that inhibiting the neuroinflammation benefits NSC self‐proliferation by targeting ApoD. In addition, when neurons were cultured in media diluted 1:1 with supernatant of LPS‐stimulated BV2 cells or microglia from AD mouse models for 48 h, FCM analysis showed that RAP treatment (20 μg/μL) dramatically inhibited the apoptosis of neurons in contrast to the control (Figure 4E,F). In summary, these data demonstrate that activated microglia construct a NLRC4 inflammasome‐associated cytokine microenvironment promoting AD progression.

## 4 DISCUSSION

AD has been identified as the most common cause of dementia, a general condition involving memory impairment and other cognitive dysfunction serious enough to affect daily life.^3^ Patients with AD account for 65%–80% of dementia cases. A well‐known risk factor is increasing age, and a majority of patients with AD are 65 years old and even older. However, Alzheimer's is not necessarily a normal part of aging or a disease of old age.^34^ AD has emerged as the sixth leading cause of death in the United States.^35^ Approximately 200 000 Americans under the 65 years old suffer from early‐onset Alzheimer's,^36^ which then worsens over time. AD has long been recognized as a progressive disease, with dementia symptoms gradually worsening over several years. In the early stage, the MCI stage, the patients present with mild memory loss. In the AD stage, patients significantly lose the ability to carry on a conversation and respond to their environments.^2^, ^37^ On average, patients with AD live 4–8.5 years after first diagnosis, but individual cases can live as long as 20 years depending on other factors. Consistent with these previous findings, our current study found that age and risk factors mainly affected the prognosis of AD patients. However, in China, there is still a shortage of big data and extensive databases to comprehensively analyze the features of Chinese AD patients, which has prompted us to develop an associated system for improving the diagnosis and treatment.

Currently, AD is at the forefront of biological medical research and scientists have discovered many possible mechanisms of AD and other dementias. Some of the most remarkable advances have shed light on how Alzheimer's pathogenesis affects brain function. Numerous potential approaches are currently under investigation around the world, with the hope of better understanding the molecular basis of the disease. Potentially, there are two abnormal structures named plaques and tangles damaging the neurons.^38^ The plaques are deposits of a protein fragment that builds up in the spaces between neurons and other stromal cells. Tangles are twisted fibers of another protein that mainly assembles inside neurons.^39^ These two structures lead to chronic inflammation, which is the main reason for AD progression. In our study, with the goal of studying the proinflammatory phenotype of microglia, we used LPS to stimulate the cells and mice. Additionally, microglia activation can be also induced by a 40–42 amino acid peptide (β‐amyloid peptide, Aβ), arising from the sequential proteolytic processing of the Amyloid Precursor Protein (APP) by beta‐ and gamma‐secretases, especially in AD models.^40^ Although the studies of Alzheimer's neuroinflammation show that many patients develop some plaques and tangles as they age, those with AD tend to develop far more and in a predictable pattern, beginning in the areas important for memory before spreading to other neural functional areas.^30^, ^39^ Even though researchers do not yet have a precise understanding of the role of neuroinflammation in AD, they believe that it blocks communications among nerve cells and disrupts the processes of proliferation and survival.

Microglia represent an intrinsic and dynamic immune cell population in CNS, which commonly secrete cytokines and phagocytose pathogenic substances. Microglial activation is the principal component of neuroinflammation and has a dual mode of action, exerting both detrimental and beneficial influences on disorders.^41^, ^42^ Well‐balanced neuronal‐microglial interactions are essential for brain function. Through their effect on stroma components, microglia are also involved in brain tumor proliferation and migration.^43^ Related to this, identification of microglial features will provide a systematic method for fine‐tuning the treatment of CNS diseases. Activated microglia are a common pathological feature of AD. Cumulative evidence has suggested that microglial inflammation in AD brain tissues is increased while microglial‐mediated clearance mechanisms are compromised.^29^ Recently, the Amit I group described a novel microglia type associated with neurodegenerative diseases by using single‐cell transcripts.^44^ In the current study, we demonstrated that microglia were activated in AD brain tissues. Specifically activated microglia presented the proinflammatory phenotype, secreting NLRC4 inflammasome‐induced IL6 and TNF‐α to promote the progression of Alzheimer's. These findings highlight the essential roles of microglia in neuroinflammation, and further offer more opportunities for therapeutic strategy.

In the current study to investigate the mechanism behind the microglial proinflammatory phenotype, we observed activation of NLRC4 inflammasomes by analyzing mRNA and protein expression. To further detect the molecular basis of inflammasome activation, we manipulated the microglia using shRNA technology to reveal the critical role of ApoD in triggering inflammasome‐mediated cytokine production. An inflammatory microenvironment is considered a hallmark of AD.^45^ Our findings indicate the important effects of ApoD on controlling microglial inflammasome activation during AD progression, and imply a general orderliness in managing the inflammatory reactions in other pathogenic conditions. However, whether inflammasome activation benefit or impair brain functions is still under debate. NSCs in hippocampus proliferate extensively, supported by other special nervous populations such as microglia, astrocytes and even mature neurons.^46^ Nonetheless, the molecular basis underlying the interactions and cell activation is not well defined. Recently, an explosion of studies has provided insights into the involvement of newly discovered non‐pathological ‘functional’ signalosome complexes in microenvironmental inflammation, such as inflammasomes, necrosomes and innateosomes.^47^, ^48^

The current findings suggest that targeting microglia activation and neuroinflammation could be a promising avenue for AD treatment. Inhibiting the overactive immune response in the brain may help mitigate AD‐related damage. These inflammatory markers could potentially serve as diagnostic or prognostic indicators in AD. Monitoring their levels could aid in early detection and tracking of disease progression. Understanding the role of ApoD in AD pathogenesis could open up new avenues for treatment. Developing therapies that modulate ApoD expression or activity may help regulate microglial inflammation. Therapies aimed at blocking ApoD or its downstream effects could potentially protect neurons from AD‐related damage. Promoting NSC self‐renewal and protecting neurons from apoptosis could be valuable therapeutic goals in AD treatment. In conclusion, our studies indicate the crucial role of ApoD in microglial inflammasome activation, and demonstrate that microglia‐induced inflammation promotes AD progression. We have established the cellular basis for microglia activation in AD progression, which sheds lights on cellular interactions important for AD treatment.

Some limitations exist in the current study. (1) Limited sample size and generalizability: the study included a relatively small sample size of 6 healthy controls and 62 cases with mild cognitive impairment (MCI) and AD. This limited sample size may not fully represent the heterogeneity of AD patients. Additionally, the study's findings may not be directly generalizable to broader populations due to the small and specific sample pool. (2) Lack of causality: while the study provides evidence of microglia activation and the involvement of NLRC4 inflammasome in AD, it does not establish causality. The observed correlations between microglial activation, NLRC4 inflammasome activation, and neuroinflammation do not prove that these mechanisms are the sole drivers of AD progression. Other factors and mechanisms may also be at play, and further research is needed to establish causative relationships. (3) Animal and cell culture models: the study relies on animal and cell culture models to investigate the mechanisms involved in AD. While these models can provide valuable insights, they may not fully recapitulate the complexity of human AD pathology. The translation of findings from these models to human patients should be done cautiously, and clinical relevance may require additional confirmation in human studies. It is essential to acknowledge these limitations when interpreting the study's results and to recognize that further research, including larger and more diverse human cohorts, longitudinal studies, and additional mechanistic investigations, are needed to gain a comprehensive understanding of the role of microglia, the NLRC4 inflammasome, and ApoD in AD.

## AUTHOR CONTRIBUTIONS

Drs. Jianzhou Lv and Yaliang Yu contributed to the whole conception and design of this project. Drs. Yaliang Yu, Ma D and Zhang YH were responsible for the details of experimental performance. Drs. Ya Han, Shanlong Wang and Jianzhou Lv made much efforts to the pathological estimates. Drs. Yaliang Yu, Xu PL and Shanlong Wang contributed to the analysis and interpretation of data. Drs. Yaliang Yu and Zhitao Wang analyzed the clinical data. Drs. Yaliang Yu and Jianzhou Lv completed the manuscript, figures and tables.

## FUNDING INFORMATION

This study was supported by the Guiding Science and Technology Development Grant in the Social Sector of Luoyang (2101083A).

## CONFLICT OF INTEREST STATEMENT

The authors declare no competing interests.

## ETHICS STATEMENT

This procedures were approved by the Research Ethics Committee of The Second Affiliated Hospital of Henan University of Science and Technology. All the methods were carried out in accordance with relevant guidelines and regulations. The study is reported in accordance with ARRIVE guidelines.

## Data Availability

The datasets used and/or analyzed during the current study available from the corresponding author on reasonable request.
